# Biopsychosocial factors associated with chronic low back pain-related activity limitations in Burundi

**DOI:** 10.4102/sajp.v78i1.1783

**Published:** 2022-07-21

**Authors:** Ildephonse Nduwimana, Félix Nindorera, Alexis Sinzakaraye, Yannick Bleyenheuft, Jean-Louis Thonnard, Oyene Kossi

**Affiliations:** 1 Institute of Neuroscience, Catholic University of Louvain, Brussels, Belgium; 2 National Reference Centre for Physiotherapy and Medical Rehabilitation (CNRKR), Bujumbura, Burundi; 3 Department of Physical Medicine and Rehabilitation, Kamenge University Hospital Centre, Bujumbura, Burundi; 4 Neurorehabilitation Unit, Neurology Department, University Hospital of Parakou, Parakou, Benin; 5 National School of Public Health and Epidemiology, University of Parakou, Parakou, Benin

**Keywords:** biopsychosocial factors, chronic low back pain, activity limitations, Burundi, cross-sectional study

## Abstract

**Background:**

Chronic low back pain (CLBP) is an increasing burden worldwide. The biopsychosocial factors associated with CLBP-related activity limitations have not yet been investigated in Burundi.

**Objective:**

The aim of our study was to investigate the biopsychosocial factors that influence the CLBP-related activity limitations in a Burundian sample population.

**Method:**

We carried out a cross-sectional study of 58 adults with nonspecific CLBP from Bujumbura city. Univariate and bivariate analyses were used to investigate the association between biopsychosocial factors and CLBP activity limitations. Sequential multiple regression analyses were subsequently used to predict CLBP activity limitations.

**Results:**

Fifty-eight individuals with a mean age of 41.3 ± 10.20, 58.6% of female gender, were recruited. The univariate and bivariate analyses demonstrated that educational level, gender, healthcare coverage, profession, height, pain intensity, depression and physical fitness were significantly associated with CLBP-related activity limitations (*p* range, < 0.001 to < 0.05). The multivariate regression analysis showed that the significant biopsychosocial factors accounted for 49% of the variance in self-reported activity limitations. Predictors of activity limitations were education level (β = −0.369; *p* = 0.001), abdominal muscle endurance (β = −0.339; *p* = 0.002) and depression (β = 0.289; *p* = 0.011).

**Conclusions:**

Our study provides evidence of biopsychosocial factor associations with CLBP-related activity limitations in Burundi. Evidence-based management and prevention of CLBP in Burundi should incorporate a biopsychosocial model.

**Clinical implications:**

Biopsychosocial factors should be regularly evaluated in people with chronic low back pain and efforts to improve the burden of chronic low back pain in Burundi should take these factors into account.

## Introduction

Low back pain (LBP) is the leading cause of disability and accounts for the most years lived with a disability worldwide (Vos et al. 2016; Wu et al. 2020). According to linear-fit estimates based on data from the last 20 years, the incidence, prevalence and disability-adjusted life years of LBP are expected to increase by ~1.4-fold by the year 2050 (Mattiuzzi, Lippi & Bovo 2020).

Population ageing and population growth are contributing to the increasing global burden of LBP, particularly in low- and middle-income countries that have overburdened health and social systems and that are less equipped to support disabled citizens (Hartvigsen et al. 2018; Meucci, Fassa & Faria 2015). The lifetime, annual and point prevalence rates of LBP in Africa are higher than the average global LBP prevalence rates (Morris et al. 2018). In a 2015 systematic review (Meucci et al. 2015) examining the worldwide prevalence of chronic low back pain (CLBP) in particular, the authors found that 4.2% of adults 24–39 years of age and 19.6% of adults 20–59 years of age suffer from CLBP.

Aetiologically, CLBP has been associated with psychosocial and biophysical factors, comorbidities and pain-processing mechanisms (Hartvigsen et al. 2018). Biopsychosocial modeling, which considers biological, psychological and social factors and their interactions, is valuable for providing information that can be used to promote good health and prevent illness (Gatchel 2004). Several studies (Alotaibi, Das Nair & Radford 2016; Fujii & Matsudaira 2013; Igwesi-Chidobe et al. 2017a; Jonsdottir et al. 2019; Kwon et al. 2006), mostly from high- and middle-income countries, have investigated the biopsychosocial factors associated with the development of CLBP. Variance in the findings amongst these studies may reflect study sample differences in culture and beliefs, as well as national differences in social and health systems (Singh et al. 2018). Biopsychosocial factors that have been associated with CLBP risk include age, being female, a low level of education, a high body mass index (BMI), a sedentary lifestyle, smoking, anxiety, depression, fear avoidance beliefs, maladaptive perceptions of illness, lack of social support and work-related dimensions (Alotaibi et al. 2016; Fujii & Matsudaira 2013; Igwesi-Chidobe et al. 2017a, 2017b, Jonsdottir et al. 2019; Kwon et al. 2006; Tarimo & Diener 2017).

Very few studies (Igwesi-Chidobe et al. 2017a, 2017b, Tarimo & Diener 2017) have investigated biopsychosocial factors associated with CLBP-related activity limitations in Africa. No such studies have been carried out in Burundi specifically. The aim of our study was to investigate the biopsychosocial factors associated with self-reported activity limitations related to CLBP in a Burundian population sample with the long-term goal of contributing to the improvement of CLBP management in Burundi.

## Methods

We conducted a cross-sectional study in Bujumbura, the largest city and economic capital of Burundi. We collected data from patients diagnosed with CLBP at a single point in time.

Recruitment started in September of 2019. The enrollment and assessment procedures took place at the Centre National de Référence en Kinésithérapie et Réadaptation Médicale (CNRKR), Bujumbura, in Burundi. Data collection started in November 2019 and was completed in February 2020. Volunteer participants were recruited through advertisements about our study and by consulting admission records of hospitals and rehabilitation centres in Bujumbura. The first author (I.N.) screened potential participants by phone. Those who were eligible and interested made appointments to come to the CNRKR, where they signed a consent form before undertaking the study assessments.

Participants were included in our study if they had a medical diagnosis confirmation of nonspecific CLBP, were 18–65 years old, resident in Burundi and had sufficient mobility to enable walking and sit-to-stand transition without assistance. Participants were excluded if they were pregnant, had any signs of serious spinal pathology (e.g. cancer, cauda equina lesion), radicular pain indicative of nerve root compression, severe spinal stenosis, spondylolisthesis and fibromyalgia.

### Data collection and variables descriptions

The first author (I.N.) and two clinicians conducted the evaluations. Before data collection, the evaluators were trained to standardise instrument administration. Because some participants lacked reading fluency, the assessments were interviewer administered. Biopsychosocial variables and CLBP-related activity limitations were collected.

#### Biopsychosocial factors

Social factors such as marital status, profession, education level and healthcare coverage were collected.

**Depression:** Chronic low back pain-associated depression was measured with the Beck Depression Inventory (BDI)-II, a 21-item questionnaire designed to measure the severity of depression. Each item references a symptom in four statements worth 0, 1, 2 and 3 points, respectively; the respondent chooses the statement that best describes the severity of his or her symptoms over the past 2 weeks (Wang & Gorenstein 2013). Beck Depression Inventory-II scores (range, 0–63) were obtained by summing the number of each marked statement and were interpreted as follows: < 10, no or minimal depression; 10–18, mild to moderate depression; 19–29, moderate to severe depression; and 30–63, severe depression (Beck Steer & Carbin 1988).

**Fear avoidance beliefs:** The Fear-Avoidance Beliefs Questionnaire (FABQ) was used to measure how participants’ fear avoidance beliefs about physical activity and work may affect their LBP. The FABQ consists of 16 items in which agreement with the statement in each item is rated on a 7-point Likert scale (0 = completely disagree, 6 = completely agree). The FABQ consists of a four-item physical activity subscale (FABQ-PA; maximum score 24), a seven-item work subscale (FABQ-Work; maximum score 42) and five additional informational or categorical items (Waddell et al. 1993).

**Biological and physical factors:** Age, gender, weight, height and BMI data were collected.

The Shirado test measures the static endurance of the abdominal muscles. The participants lay in a supine position with their hips and knees bent at 90°, calves resting on a stool or chair and arms crossed over the chest with their hands resting on their shoulders. They were asked to lift their scapulae off the floor and maintain this crunch position as long as possible (Ito et al. 1996). We noted the hold time in seconds without encouraging the subject or specifying the time during the test. The test ended for each participant when his or her scapulae touched the floor because of fatigue or discomfort or after a maximum duration of 240 s.

The Sorensen test assesses the endurance of the trunk extensor muscles. The participants lay on the examining table in the prone position with the upper edge of the iliac crests aligned with the edge of the table. Their lower bodies were fixed to the table, their arms folded across their chest and they were asked to maintain their upper bodies in a horizontal position isometrically (Biering-Sørensen 1984; Demoulin et al. 2006). The time during which each participant held his or her upper body straight and horizontal was recorded. The test was stopped if the trunk dropped because of fatigue or discomfort or after a maximum duration of 240 s. The Shirado test was performed before the Sorensen test for all patients, and the two tests were separated by a rest interval in order to decrease muscle fatigue-related measurement bias.

Borg’s Category Ratio-Scale (Borg CR10) was used to assess patient-perceived exertion (Borg 2008). Borg CR10 is a common scale used to assess sensory perceptions, subjective symptoms and feelings. We used Borg CR10 to assess the exertion perception and therefore the aerobic capacity of the participants. The Borg CR10 was completed at the end of each stage of a step test by asking the participants to rate their perception of physical effort or exertion after 2 min of stepping up and down. The step test (Nielens & Plaghki 1991) was organised according to submaximal, gradational and discontinuous principles. We used four wooden platforms of different heights (0.07, 0.12, 0.19 and 0.24 m). Patients were instructed to step up and down for 2 min at a frequency of 84 steps/min (given by a metronome). Patients were allowed to rest seated on a chair for 2 min after each stage. They were encouraged to perform as many stages as they could.

Participants were asked to report the intensity of their pain on an 11-point numerical rating scale (NRS) ranging from 0 to 10, wherein 0 represented the absence of pain and 10 represented the worst pain imaginable (Hawker et al. 2011). In accordance with Jensen et al.’s recommendations, we adopted the following NRS interpretation rubric: 1–4, mild pain; 5–6, moderate pain; and 7–10, severe pain (Jensen Chen & Brugger 2003).

### Chronic low back pain-related activity limitations

Activity limitations were measured with the Roland Morris Disability Questionnaire (RMDQ). The RMDQ consists of 24 items that refer to different ways that LBP may affect activities. Participants ticked the limitation items that applied to themselves. The RMDQ scores were the number of ticked items, such that higher RMDQ scores indicate greater activity limitations and thus greater disability (Roland & Morris 1983).

### Data analysis

The data were analysed in SPSS version 27 software. For each study variable, the normality of data was verified by means of histograms and Q–Q plots. Descriptive statistics were used to present the study sample’s biopsychosocial and CLBP activity limitations. Outcomes are reported as means with standard deviations for continuous and normally distributed variables, medians with interquartile ranges for ordinal and non-normally distributed variables and numbers of cases with percentages for nominal variables. Univariate analyses were done to determine the association of biopsychosocial variables with disability. A *t*-test and one-way Analysis of Variance (ANOVA) were used to investigate the association of nominal biopsychosocial factors with the CLBP activity limitations measure. Pearson’s and Spearman’s correlation coefficients were used to investigate the bivariate relationship between activity limitations measure and quantitative biopsychosocial factors, according to the normality of data. Correlation coefficient values were reported with *p*-values and interpreted as follows (Schober, Boer & Schwarte 2018): 0.0–0.10, negligible; 0.10–0.39, weak; 0.40–0.69, moderate; 0.70–0.89, strong; and 0.90–1.0, very strong. The association of each biopsychosocial variable with RMDQ was tested for inclusion in the multivariable analysis using a significance level of *p* < 0.2. Stepwise linear regression was used to reveal the most important predictors. The stepwise linear regression iteratively examined the statistical significance of each independent variable and selected significant biopsychosocial factors to be used in the final model. The assumption of collinearity was assessed by inspection of tolerance and Variance Inflation Factor (VIF) values. Unstandardised regression coefficient (B), standardised coefficient (Beta), *t*-, *p*-value and 95% confidence interval for B were reported for each model to demonstrate the relative importance of individual predictors. The significance level for retaining a factor in the final stepwise multiple regression model was set at *p* < 0.05.

### Ethical considerations

Ethical clearance to complete the research was obtained from the National Ethics Committee for the Protection of Human Subjects of Biomedical and Behavioral Research (reference numbers: CNE/20/2019; 12/12/2019).

## Results

### Characteristics of the study participants

**FIGURE 1:**
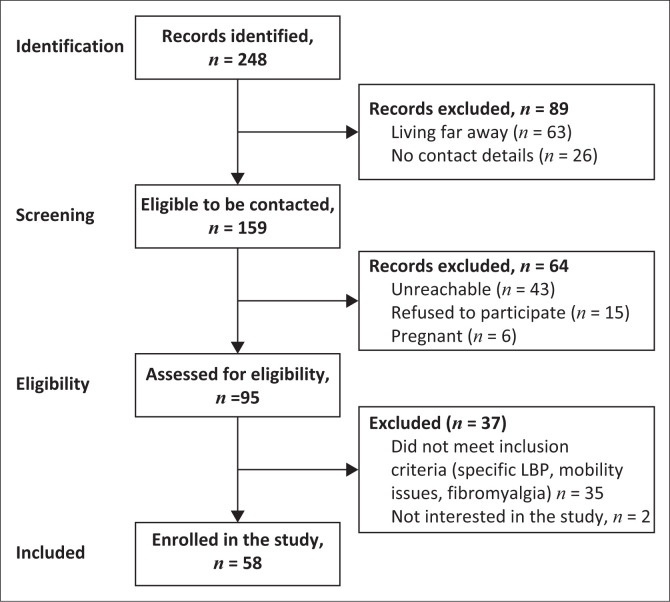
Study flowchart.

A total of 248 individuals with LBP were identified in the hospital admission registries and rehabilitation settings. After inclusion and exclusion criteria application, 58 participants were included in our study. Figure 1 shows the process for the selection of the participants. The characteristics of the 58 study participants with CLBP in terms of biopsychosocial factors and activity limitations are presented in Table 1. Notably, the majority of the sample were female, married, employed and with a university-level education. Most of the participants had minimal depression (median BDI-II score = 6) without a gender difference.

**TABLE 1:** Chronic low back pain-related activity limitations and biopsychosocial outcomes, *n* = 58.

Variables	*n*	%	Mean ± s.d./median [25; 75]
**Gender**			
Female	34	58.6	-
Male	24	41.4	-
**Marital status**			
Married	43	74.1	-
Single	12	20.7	-
Divorced	1	1.7	-
Widowed	2	3.4	-
**Profession**			
Employee	39	67.2	-
Self-employed	9	15.5	-
Unemployed	10	17.2	-
**Education level**			
No university	21	36.2	-
University	37	63.8	-
**Healthcare coverage**			
Yes	42	72.4	-
No	16	27.6	-
Age (years)	-	-	41.3 ± 10.20
Weight (kg)	-	-	72.4 ± 13.15
Height (cm)	-	-	1.65 ± 0.08
Body mass index (kg.m^−2^)	-	-	26.5 ± 4.44
Shirado test	-	-	25.0 ± 25.72
Sorensen test	-	-	42.3 ± 45.47
Borg CR10	-	-	4.1 ± 2.62
BDI	-	-	7.8 ± 6.06
FABQ-PA	-	-	7.0 [0.00; 15.00]
FABQ-Work	-	-	12.5 [4.00; 22.00]
NRS	-	-	6.6 ± 1.96
RMDQ	-	-	10.3 ± 4.25

Borg CR10, Borg Category Ratio Scale; BDI, Beck Depression Inventory; FABQ-PA, Fear Avoidance Beliefs Questionnaire-Physical Activity; FABQ-Work, Fear Avoidance Beliefs Questionnaire-Work; NRS, Numerical Rating Scale; RMDQ, Roland and Morris Disability Questionnaire; 5-IPDI, 5-Item Pain Disability Index; s.d., standard deviation.

### Association of biopsychosocial factors and chronic low back pain-related activity limitations

The results of our analyses of the significant relationships of categorical biopsychosocial factors with RMDQ are reported in Table 2. Compared with participants who had a university education, participants with less than a university level of education had higher activity limitations (RMDQ scores; *p* < 0.001). On average, women had more activity limitations (RMDQ; *p* < 0.01) than men. Participants without healthcare coverage had more activity limitations (RMDQ; *p* < 0.05) than participants with healthcare coverage.

**TABLE 2:** Chronic low back pain-related activity limitations distribution according to sociodemographic variables (*n* = 58).

Variables	Roland and Morris Disability Questionnaire		*p*
	Mean	± Standard deviation	
**Education level**			< 0.001
No university (*n* = 21)	13.3	± 4.35	
University (*n* = 37)	8.6	± 3.17	
**Gender**			< 0.01
Male (*n* = 24)	8.6	± 3.61	
Female (*n* = 34)	11.5	± 4.30	
**Healthcare coverage**			< 0.05
Yes (*n* = 42)	9.3	± 3.43	
No (*n* = 16)	12.9	± 5.14	
**Marital status**			0.494
Single (*n* = 43)	10.8	± 4.50	
Married (*n* = 12)	8.7	± 3.44	
Divorced (*n* = 1)	-		
Widowed (*n* = 2)	10.0	± 1.41	
**Profession**			< 0.05
Employed (*n* =39)	9.4	± 3.22	
Self-employed (*n* = 10)	12.2	± 5.71	
Unemployed (*n* = 9)	12.4	± 5.34	

*n*, Number of subjects.

Table 3 shows the bivariate Pearson’s and Spearman’s correlation coefficients between activity limitations (RMRQ) and the biopsychosocial variables. Roland Morris Disability Questionnaire scores (higher scores indicate greater activity limitations) correlated directly with pain intensity (ρ = 0.36; *p* < 0.01), depression (ρ = 0.48; *p* < 0.01) and exertion perception (ρ = 0.45; *p* < 0.01), whilst correlating inversely with height (ρ = −0.42; *p* < 0.01) and muscle endurance measures (Shirado test, ρ = −0.46; *p* < 0.01; Sorensen test ρ = −0.52; *p* < 0.01).

**TABLE 3:** Pearson and Spearman correlation between low back pain disability and biopsychosocial factors (*n*=58).

Variables	Age	Weight	Height	BMI	NRS	BDI	FABQ-PA	FABQ-Work	SHIRADO test	SORENSEN test	Borg CR10
RMDQ	0.036	−0.039	−0.418**	0.223	0.365**	0.478**	0.104	0.095	−0.463**	−0.517**	0.455**
Age	-	0.139	0.211	−0.020	−0.226	−0.327*	−0.078	−0.082	0.103	0.190	−0.196
Weight	-	-	0.437**	0.826**	−0.021	−0.120	0.005	0.106	−0.098	−0.045	0.034
Height	-	-	-	−0.120	−0.191	−0.323*	−0.121	0.261	0.329*	0.319*	−0.382**
BMI	-	-	-	-	0.109	0.098	0.119	−0.033	−0.320*	−0.236	0.318*
NRS	-	-	-	-	-	0.311*	0.146	0.224	−0.259*	−0.461**	0.339*
BDI	-	-	-	-	-	-	0.216	0.361*	−0.192	−0.341**	0.315*
FABQ-PA	-	-	-	-	-	-	-	−0.083	0.040	−0.156	0.003
FABQ-Work	-	-	-	-	-	-	-	-	−0.219	−0.163	0.248
SHIRADO test	-	-	-	-	-	-	-	-	-	0.543**	−0.457**
SORENSEN test	-	-	-	-	-	-	-	-	-	-	−0.563**

RMDQ, Roland and Morris Disability Questionnaire; BMI, Body Mass Index; NRS, Numerical Rating Scale; BDI, Beck Depression Inventory; FABQ-PA, Fear Avoidance Beliefs Questionnaire-Physical Activity; FABQ-Work, Fear Avoidance Beliefs Questionnaire-Work; Borg CR10, Borg Category Ratio Scale.

* , correlation is significant at the 0.05 level (2-tailed).

** , correlation is significant at the 0.01 level (2-tailed).

Pearson correlation: RMDQ, age, weight, height, BMI, NRS, BDI, SHIRADO test, SORENSEN test, and Borg CR10.

Spearman correlation: FABQ-PA, FABQ-Work.

Table 4 shows the stepwise multiple regression analysis predicting self-reported activity limitations. All biopsychosocial variables associated with RMDQ (Table 2 and Table 3), with a significance level of *p* < 0.2, were entered into the model to control their effect. This model explained 49% of the variance in RMDQ (*R*^2^ = 0.49). The significant predictors of RMDQ were education level (β= −0.369; *p* = 0.001), abdominal endurance (β = −0.339; *p* = 0.002) and depression (β = 0.289; *p* = 0.011).

**TABLE 4:** Results of stepwise multiple linear regression model for the association between biopsychosocial variable and activity limitations related to chronic low back pain in Burundi (*n* = 58).

Variable	*B*	s.e.	Beta	*t*	*p*	95% confidence interval
Constant	15.433	1.919	-	8.043	0.000	11.577 to 19.290
Education level	−3.229	0.958	−0.369	−3.370	0.001	−5.155 to −1.304
Shirado test	−0.056	0.017	−0.339	−3.231	0.002	−0.091 to −0.021
BDI	0.203	0.077	0.289	2.640	0.011	0.048 to 0.357

BDI, Beck Depression Inventory; s.e., standard error.

## Discussion

Our study investigated biopsychosocial factors that may influence self-reported activity limitations related to CLBP in a Burundian population sample. The most important predictors of CLBP-related activity limitations in Burundi were education level, abdominal endurance and depression. The model with these three predictors explained 49% of the variance in self-reported activity limitations.

Our findings support previous evidence in high- and middle-income countries, suggesting that a low level of education is associated with a risk of LBP and associated functional limitations (Batista Henschke & Oliveira 2017; Dionne et al. 2001; Reisbord & Greenland 1985). These associations could reflect variations in behavioural and environmental risk factors as well as variations in living and work conditions. For example, people without a university education are more likely to have occupations that are associated with lumbar spine strain and are less likely to have adequate access to health services and to have developed adaptive stress-coping strategies (Meucci et al. 2015; Reisbord & Greenland 1985). In contrast, highly educated persons are more likely to be in professional, managerial or other skilled occupations that are generally less physically demanding and where there is more flexibility to eliminate pain-provoking occupation situations (Haber 1973).

Our data showing that depression predicted self-reported activity limitations are similar to previous publications (Hung, Liu & Fu 2015; Nassar et al. 2019) reporting that depression is an important factor associated with disability amongst patients with CLBP. Hung et al. (2015) explained this association by negative thoughts, fatigue and lack of motivation and self-confidence, which are common symptoms of depression. These depressive symptoms might decrease physical activities. Furthermore, patients with depression might underestimate their abilities and daily function (Huijnen et al. 2010). Then, a depressed mood can distort a patient’s view on his or her own activities. However, the directionality of the relationship between depression and CLBP is unclear and could be complex. In fact, Probst et al. (2016) suggested that depression mediated the effect pain exerts on disability. According to Rush, Polatin and Gatchel (2000) and Ma et al. (2021), chronic diseases may lead to depression, and depression may also lead to chronic diseases.

The physical fitness-related data analysis results support the notion that good physical fitness may be protective against CLBP. Specifically, we found that low measures of isometric abdominal muscle endurance predicted high activity limitations. Our results are similar to the findings of Abdelraouf and Abdel-Aziem (2016) and Correia et al. (2016), who report that poor abdominal endurance is associated with LBP in collegiate male athletes and tennis players, respectively. Bezgin et al. (2020) found a significant relationship between balance, trunk muscular endurance and functional level in individuals with CLBP. Moreover, Da Silva et al. (2015) conclude that individuals with nonspecific LBP presented with significant lumbar musculature fatigue compared with people without nonspecific LBP, both in younger and older adults. As trunk muscles are active in most daily activities, getting tired will prevent the patients from performing their activities throughout the day (Bozorgmehr et al. 2018).

Although gender and healthcare coverage are no longer associated with activity limitations in the multiple regression model, these variables would seem to have an impact in the African context. Despite changes in traditional gender roles and a general increase in participation in housework by men, women still perform a greater amount of household work (Fehrmann et al. 2019). This seems to be more pertinent in the African context. Furthermore, women are likely more physically inactive during leisure time, probably because of their dual role (Harreby et al. 1997; Sheffer et al. 2002).

Only 22.0% of the Burundian population has healthcare coverage (Barasa et al. 2021). We found that low healthcare coverage in Burundi was associated with activity limitations. Levels of healthcare coverage frequently determine the type of care received for certain medical conditions. The lack of healthcare services patients need may explain CLBP-related activity limitations’ association with healthcare coverage (Enyinnaya, Taylor & Knaus 2012). This finding underscores the need for potentially less costly treatment options to best fit the needs of individuals with no or low healthcare coverage (Enyinnaya et al. 2012).

### Study strength and limitations

Our work is the first cross-sectional study to demonstrate associations of biopsychosocial factors with CLBP-related activity limitations in Burundi. However, some biopsychosocial factors that have been reported to be associated with CLBP disability in other populations and thus could be relevant in the Burundian population were not investigated in our study, including self-efficacy, illness perception, catastrophising, coping strategies, work absenteeism and isolation, amongst others. Furthermore, the small sample size may not be representative of the Burundian population and limits the generalisability of our study finding.

## Conclusion

We found that CLBP-related activity limitations were significantly associated with biopsychosocial factors, including education level, depression and abdominal muscle endurance in Burundian patients. These biopsychosocial factors have been found to be associated with CLBP-related activity limitations in high- and middle-income countries. These findings support the incorporation of a biopsychosocial model in evidence-based prevention and management of CLBP in Burundi. Future studies of CLBP in Burundi should examine larger samples and investigate more biopsychosocial factors.
